# Feasibility, reliability and validity of health-related quality of life questionnaire among adult pulmonary tuberculosis patients in urban Uganda: cross-sectional study

**DOI:** 10.1186/1477-7525-8-93

**Published:** 2010-09-02

**Authors:** Harriet M Babikako, Duncan Neuhauser, Achilles Katamba, Ezekiel Mupere

**Affiliations:** 1 School of Public Health, College of Health Sciences, Makerere University P O Box 7072 Kampala, Uganda; 2 Department of Epidemiology and Biostatistics, Case Western Reserve University, 10900 Euclid Avenue, 44106 Cleveland Ohio, USA; 3 Clinical Epidemiology Unit, Department of Internal Medicine, School of Medicine, Makerere University P O Box 7072 Kampala, Uganda; 4 Department of Paediatrics and Child Health, School of Medicine, Makerere University P O Box 7072 Kampala, Uganda

## Abstract

**Background:**

Despite the availability of standard instruments for evaluating health-related quality life (HRQoL), the feasibility, reliability, and validity of such instruments among tuberculosis (TB) patients in different populations of sub-Saharan Africa where TB burden is of concern, is still lacking.

**Objective:**

We established the feasibility, reliability, and validity of the Medical Outcomes Survey (MOS) in assessing HRQoL among patients with pulmonary tuberculosis in Kampala, Uganda.

**Methods:**

In a cross-sectional study, 133 patients with known HIV status and confirmed pulmonary TB disease were recruited from one public and one private hospital. Participants were enrolled based on duration of TB treatment according to the following categories: starting therapy, two months of therapy, and eight completed months of therapy. A translated and culturally adapted standardized 35-item MOS instrument was administered by trained interviewers. The visual analogue scale (VAS) was used to cross-validate the MOS.

**Results:**

The MOS instrument was highly acceptable and easily administered. All subscales of the MOS demonstrated acceptable internal consistency with Cronbach's alpha above 0.70 except for role function that had 0.65. Each dimension of the MOS was highly correlated with the dimension measured concurrently using the VAS providing evidence of validity. Construct validity demonstrated remarkable differences in the functioning status and well-being among TB patients at different stages of treatment, between patients attending public and private hospitals, and between men and women of older age. Patients who were enrolled from public hospital had significantly lower HRQoL scores (0.78 (95% confidence interval (CI); 0.64-0.95)) for perceived health but significantly higher HRQoL scores (1.15 (95% CI; 1.06-1.26)) for health distress relative to patients from private hospital. Patients who completed an 8 months course of TB therapy had significantly higher HRQoL scores for perceived health (1.93 (95% CI; 1.19-3.13)), health distress subscales (1.29 (95% CI; 1.04-1.59)) and mental health summary scores (1.27 (95% CI; 1.09-1.48)) relative to patients that were starting therapy in multivariable analysis. Completion of 8 months TB therapy among patients who were recruited from the public hospital was associated with a significant increase in HRQoL scores for quality of life subscale (1.26 (95% CI; 1.08-1.49)), physical health summary score (1.22 995% CI; 1.04-1.43)), and VAS (1.08 (95% CI; 1.01-1.15)) relative to patients who were recruited from the private hospital. Older men were significantly associated with lower HRQoL scores for physical health summary score (0.68 (95% CI; 0.49-0.95)) and VAS (0.87 (95% CI; 0.75-0.99)) relative to women of the same age group. No differences were seen between HIV positive and HIV negative patients.

**Conclusion:**

The study provides evidence that the MOS instrument is valid, and reliably measures HRQoL among TB patients, and can be used in a wide variety of study populations. The HRQoL differed by hospital settings, by duration of TB therapy, and by gender in older age groups.

## Background

In Uganda, the estimated overall tuberculosis (TB) incidence is 411 cases per 100,000 population and ranks 16^th ^among the 22 high-burden countries for TB. The Uganda TB treatment success (68%) is far below the WHO target of 85% [1,2]. A comprehensive understanding of barriers to and facilitators of poor TB treatment outcome is still lacking, and this is a major obstacle to finding effective solutions. The current TB program services and clinical research have focused on outcomes of mortality and microbiologic cure, and have neglected patient's preferences such as patient's perceived health-related quality of life (HRQoL) which may be crucial in influencing treatment outcome. Health-related quality of life involves assessing a person's perception of his or her physical and mental health [3]. Both physical and mental distress is common in TB patients leading to poor disease outcome or poor treatment outcome because of decreased ability to take treatment [4,5]. Knowing patient's HRQoL would enable program managers and clinicians to understand the functioning and well being of TB patients so that individual patient specific needs are addressed to attain the best clinical or treatment outcome, and thus increasing the likelihood of adequate case management in TB programs.

Despite the availability of standard instruments for assessing HRQoL [6-8], the feasibility; reliability; and validity of such instruments among TB patients in different populations of sub-Saharan Africa, where the burden of TB is of concern, is still limited. This paper fills in this gap with results from a cross-sectional study that evaluated HRQoL among adult TB patients attending public and private program clinics in Kampala, Uganda. We hypothesized: 1) that HRQoL would be better among patients who have been longer on TB therapy than patients starting therapy; 2) that HRQoL would be better among patients attending private hospital compared to public hospital; 3) that HIV negative patients and 4) women would have better HRQoL compared to HIV positive patients and men, respectively.

## Methods

### Design and Setting

We conducted a cross-sectional study between November 2007 and Apri12008 to validate the HRQoL instrument among TB patients. The study centers were Mulago TB treatment center, located at the national teaching hospital, Mulago; and Mengo TB clinic, located at Mengo missionary hospital. Mulago a public hospital and Mengo a private hospital were chosen to achieve patient heterogeneity in the study population, and to understand how patient HRQoL differs by hospital setting. In addition, Mulago hospital was chosen because it serves the largest number of TB patients in Kampala, the capital city. Mengo hospital was conveniently chosen to represent the private missionary hospitals in Kampala city. The Mulago TB treatment center is the principal facility that provides in-patient and outpatients TB care in Kampala city. It has a bed capacity of about 100 beds. The Mulago treatment center registers more than 150 new TB patients a month while Mengo registers about 30.

All TB patients are provided with an opt-out option for HIV counseling and testing at the two hospitals. Identification of TB patients in both treatment centers is by passive case-finding as recommended by the Uganda National Tuberculosis and Leprosy Program (NTLP). Passive case-finding is self-referral of symptomatic individuals to health facilities. The main diagnostic method is sputum microscopy with two positive alcohol-fast bacilli (AFB) smear test or one positive smear test with suggestive chest X- rays findings. During care under the Uganda NTLP guideline recommendation [9], patients in this study received short course chemotherapy with daily Rifampicin, Isoniazid, Pyrazinamide, and Ethambutol (RHZE) for 2 months and during the continuation phase of 6 months with Isoniazid and Ethambutol (EH).

The protocol was approved by the Faculty of Medicine, Research Ethics Committee and the Uganda National Council for Science and Technology. Participants provided written consent.

### Subjects

Study participants18 or more years of age and identified to have confirmed new TB disease at Mulago and at Mengo TB treatment centers were eligible for recruitment into the study. Participants were consecutively and conveniently enrolled according to the following categories: starting TB treatment, completing two months of treatment, and completing eight months of treatment. Participants residing outside Kampala district or residing beyond 20 kilometers from the treatment centers were excluded. All participants spoke the local language-Luganda.

### Procedures

Identification of eligible participants and administration of the questionnaires were conducted by two study nurses. The study nurses administered the questionnaires in face-to-face interviews after the patient exited the pharmacy unit. The study questionnaires measured HRQoL, HIV status, and socio-demographic information. The study nurses were not involved in the routine care of patients at the individual clinics. Patient's HIV sero-status was obtained verbally from the individual patient and later confirmed with hospital records. Each participant was reimbursed with lunch valued at $1.50 after the interview. Data were double-entered using EpiData version 3.1 2008 [10].

The Medical Outcome Survey (MOS) was used to measure HRQoL among TB patients [11]. The MOS questionnaire had been previously translated and culturally adapted in Uganda among HIV-infected individuals [7]. The MOS results were validated using the visual analogue scale (VAS) [12,13]. We used the MOS because it has been shown to have good internal reliability in a wide variety of settings, an excellent discriminant and convergent validity of the subscales [11,14], and good physical and mental health summary scores in HIV disease [7,15]. The MOS survey consists of 35 questions which assess ten dimensions of health including general health perceptions, pain, physical functioning, role function, social functioning, mental health, energy/fatigue, cognitive function, health distress and quality of life (QoL). One of the items assesses health transition [11]. For each of the MOS subscales, responses to individual questions were aggregated and scores were converted to a 0-to-100 point scale, with 100 representing the best health status or function. Physical (PHS) and mental health (MHS) summary scores were calculated according to standard guidelines [15] to have a mean of 50 and a standard deviation of ten.

The 100 cm "feeling thermometer" was used for the visual analogue scale (VAS). Patients indicate their self-perceived quality of life for the day from 0 for the poorest imaginable state to 100 for the best imaginable health state. The interviewer first reviewed the interval properties of the scale then asked the participant to locate the health state on a 100-point scale.

### Statistical analyses

The feasibility of conducting quality of life interviews among TB patients using the MOS in urban Uganda setting was evaluated by examining the percent of missing item responses, interviewer-reported acceptability, and the time and ease of administration. Cronbach's coefficient was calculated to estimate reliability for multi-item scales. In general, coefficient ≥ 0.70 indicates satisfactory reliability [16]. Pearson coefficients were used to correlate respondent's evaluations of their own health states using the VAS and MOS.

The construct validity of HRQoL scores was evaluated in four ways: 1) the researchers hypothesized that there would be differences in the magnitude of the scores for patients starting TB therapy, completing two months on therapy, and those with completed therapy; 2) there would be differences in the magnitude of the scores for patients accessing public care services at Mulago and private care services at Mengo hospitals; 3) there would be differences in magnitude of the scores for HIV positive and HIV negative TB patients; and 4) there would be differences in magnitude of the scores for men and women. Differences between group means of the scores were compared using Wilcoxon-Mann Whitney test due to lack of normality for the scores and reduced power in subgroup analysis. Bonferroni corrections were used to adjust for multiple comparisons and a p-value of <0.008 was taken as significant. We adjusted for sex, HIV status, age and hospital setting. Differences in proportions were tested for using chi-square test.

The effect of variables such as hospital setting, sex, HIV sero-status, and age group on HRQoL scores of the MOS subscales and summary scales were calculated. The effect was calculated using multiple linear regression analysis. The scores for HRQoL of all the subscales were skewed. Therefore, a logarithmic transformation was used to make the data more normally distributed. Relative HRQoL scores by the exponential of regression coefficients from multiple regression analysis were estimated. We evaluated two-way interactions between sex and age group, patient category, or hospital setting; and between hospital setting and patient category. A p-value of less than 0.05 was considered statistically significant. All analysis was performed using SAS software version 9.2 (SAS Institute Inc., Cary, NC; 2004).

## Results

### Patient characteristics

Of the 133 participants who were enrolled into the study, 67 were recruited from the public (Mulago) and 66 from the private (Mengo) hospital (Table 1). Further, 46 were starting TB treatment, 44 had completed two months on treatment, and 43 had completed a full course of 8 months treatment. The male to female ratio was 1:1, similarly, HIV positive to HIV negative TB patients. Four patients (3%) were of unknown HIV status. There were no differences in mean age, in proportions of men, and in proportions of patient categories (i.e., starting TB therapy, two months on therapy, and eight months on therapy) between patients who were enrolled from public and private hospitals (Table 1).

**Table 1:** Characteristics of 133 tuberculosis study participants in Kampala, Uganda, 2007-2008

Characteristics		Public hospital (n = 67)	Private hospital (n = 66)
Sex			
	Men (%)	34 (51)	33 (50)
	Women (%)	33 (49)	33 (50)
HIV-serostatus^1^			
	Positive (%)	32 (49)	32 (50)
	Negative (%)	33 (51)	32 (50)
Mean age (years) SD^2^		32.0 ± 9.9	35.2 ± 11.2
Patient category			
	Starting therapy (%)	24 (36)	22 (33)
	Two months on therapy (%)	21 (31)	23 (35)
	Completed therapy (%)	22 (33)	21 (32)

Proportions were compared using chi-square test and means using Wilcoxon-Mann Whitney; none was significant. ^1^Four patients were of unknown HIV status. ^2^Values are means with ± standard deviation.

### Feasibility and reliability testing

There were few missing responses to the MOS; less than 1% (1/133) of the participants had missing responses for any items. The MOS took approximately 13 minutes to complete and was generally well tolerated by the participants. Interviewers reported that respondents had no difficulty understanding concepts of the MOS items. Cronbach's alpha coefficients for all subscales were >0.70 except for role function that had 0.65 (Table 2), suggesting satisfactory internal reliability in general. All MOS subscales correlated highly with the VAS scores (Table 2).

**Table 2:** Reliability of the Medical Outcomes Survey involving 133 tuberculosis participants in Kampala, Uganda, 2007-2008

Subscale	Number of items	Cronbach's α	Correlation with VAS score^a^
Perceived health	5	0.81	0.48
Bodily pain	2	0.88	0.46
QoL	1	-^2^	0.47
Role functioning	2	0.65	0.42
Social functioning^1^	1	-^2^	0.49
Vitality	4	0.89	0.48
Mental health	5	0.90	0.47
Health distress	4	0.96	0.40
Cognitive function	4	0.88	0.35
Physical functioning	6	0.89	0.46
Health transition	1	-^2^	0.38

^a^All Medical Outcomes Survey subscales had a correlation p-value <0.001. ^1^One individual missed social function response. ^2^Internal consistency reliability cannot be calculated for a single-item scale.

### Health-related quality of life scores

**Figure 1:**
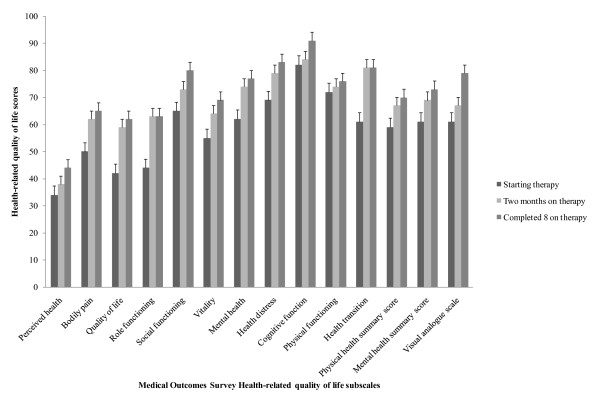
**Health-related quality life scores among adult tuberculosis patients in Kampala Uganda, 2007-2008**. Whiskers are standard errors (SEs) whereas bars represent health-related quality of life (HRQoL) scores for eleven subscales and two summary scores of the 35-item Medical Outcomes Survey (MOS) questionnaire; and HRQoL scores for the visual analogue scale (VAS) that was used to validate the MOS. The HRQoL scores were evaluated among patients starting, completing two months, and completing 8 months tuberculosis therapy. The eleven subscales of the MOS included general health perceptions, pain, quality of life, role function, social functioning, vitality (energy/fatigue), mental health, health distress, cognitive function, physical functioning, and health transition. The MOS summary scores included physical and mental health summary scores.

In general, all scores of the MOS subscales and VAS increased as the patients' duration of TB treatment increased (Figure 1). Patients with completed TB therapy had the highest magnitude of HRQoL scores regardless of the MOS subscale compared to patients starting or patients that had completed two months of therapy. For example, perceived health scores were 33.6 ± 27.7 among patients starting TB therapy, 37.7 ± 27.2 among patients with two completed months of therapy, and 43.8 ± 23.6 among patients with completed 8 months of therapy while the VAS scores were 60.7 ± 11.9, 67.1 ± 13.6, and 78.5 ± 12.8, respectively.

Of all the MOS subscales, perceived health, bodily pain, quality of life, and role function had the lowest scores (Figure 1). When multiple comparisons were made, we found significant differences in HRQoL scores between patients starting therapy and patients who had completed 8 months course of TB therapy for QoL (41.8 ± 29.4 versus 62.5 ± 26.1; p = 0.001), mental health (61.6 ± 25.5 versus 76.9 ± 20.8; p = 0.003), and health transition (60.9 ± 29.2 versus 81.3 ± 26.5; p = 0.002) MOS subscales; mental health summary score (60.5 ± 21.6 versus 73.4 ± 17.4; p = 0.006); and visual analogue scale (60.7 ± 11.9 versus 77.9 ± 13.3; p < 0.001), respectively (Figure 1).

Patients who were recruited at the public hospital had significantly lower scores of perceived general health (31.4 ± 18.2 versus 45.2 ± 31.4; p = 0.014) and VAS (65.0 ± 13.9 versus 72.2 ± 14.6; p = 0.004) compared to patients who were recruited at the private hospital, respectively (Table 3). For all the MOS subscales and the VAS, there were no significant differences between men and women, and between HIV positive and HIV negative TB patients (Table 3). A similar relationship was found in univariate linear regression analysis (Table 4).

**Table 3:** Medical Outcomes Survey subscales and mean HRQoL scores among 133 tuberculosis participants in Kampala, Uganda, 2007-2008

	Hospitals (n = 133)		Gender (n = 133)		HIV status (n = 129)^1^	
**MOS Subscale**	**Public (n = 67)**	**Private (n = 66)**	**Men (n = 67)**	**Women (n = 66)**	**HIV sero-positive (n = 64)**	**HIV sero-negative (n = 65)**

Perceived health	31.4 ± 18.2	45.2 ± 31.4^b^	40.8 ± 27.1	35.6 ± 25.6	41.3 ± 26.6	36.3 ± 26.4
Bodily pain	62.4 ± 28.5	55.0 ± 26.2	60.4 ± 29.0	57.0 ± 26.2	58.6 ± 28.5	59.0 ± 27.2
Quality of life	53.7 ± 20.5	54.5 ± 34.2	53.7 ± 27.6	54.5 ± 28.7	55.1 ± 26.8	55.4 ± 28.8
Role functioning	52.2 ± 45.6	60.6 ± 39.7	59.0 ± 43.5	53.8 ± 42.3	55.5 ± 44.6	57.7 ± 41.7
Social functioning	74.0 ± 33.0	70.6 ± 29.2	72.8 ± 31.7	71.8 ± 30.8	71.9 ± 31.6	72.0 ± 31.4
Vitality	62.1 ± 19.9	62.7 ± 24.6	63.6 ± 23.5	61.1 ± 21.0	62.8 ± 22.6	61.9 ± 22.3
Mental health	73.4 ± 19.8	67.9 ± 22.4	71.4 ± 23.9	70.0 ± 22.8	72.1 ± 24.3	69.5 ± 22.7
Health distress	84.2 ± 21.4	69.5 ± 30.8^b^	79.3 ± 26.5	74.4 ± 28.3	77.7 ± 27.5	76.1 ± 28.0
Cognitive function	86.0 ± 18.2	85.6 ± 17.4	84.5 ± 18.9	87.2 ± 16.4	83.2 ± 19.3	88.2 ± 16.1
Physical functioning	75.5 ± 20.3	71.8 ± 27.6	76.5 ± 24.0	70.8 ± 24.3	74.3 ± 23.4	73.8 ± 23.9
Health transition	71.3 ± 23.9	76.5 ± 30.0	72.8 ± 27.1	75.0 ± 27.4	75.8 ± 24.8	73.1 ± 29.7
PHS	65.4 ± 21.3	64.3 ± 25.1	67.0 ± 24.1	62.8 ± 22.2	65.1 ± 24.1	65.0 ± 22.4
MHS	67.8 ± 14.7	66.9 ± 24.8	68.2 ± 20.5	66.5 ± 20.2	68.3 ± 21.5	66.8 ± 19.7
Visual analogue scale	65.0 ± 13.9	72.2 ± 14.6^b^	69.1 ± 16.1	68.0 ± 13.1	67.6 ± 14.7	69.8 ± 14.9

^1^Four were of unknown HIV status; ^b^p-value <0.05. MOS = Medical Outcome Survey, HRQoL = Health-related quality of life. Comparisons were made using Wilcoxon-Mann Whitney test.

**Table 4:** Relative Medical Outcomes Survey HRQoL scores involving 133 tuberculosis participants in Kampala, Uganda, 2007-2008

Selected MOS Health-related quality of life subscales		variables									
		
		Hospital	Sex	HIV status	Patient category		Age group			Interactions	
		
		Public	Men	HIV positive	Two mo therapy	Complete 8 mo therapy	25-34 yrs	35-44 yrs	45+yrs	Male*35-44 yrs	Public*8 mo therapy
Perceived health	Univariate	0.76 **(0.62-0.92)**	1.32 (0.88-1.99)	1.14 (0.75-1.72)	1.01 (0.65-1.56)	1.62 **(1.06-1.22)**	0.82 (0.53-1.25)	1.22 (0.76-1.97)	1.84 **(1.05-3.22)**	-	-
	Multivariate	0.78 **(0.64-0.95)**	1.34 (0.90-2.00)	0.90 (0.58-1.39)	1.43 (0.88-2.32)	1.93 **(1.19-3.13)**	1.18 (0.68-2.05)	1.50 (0.80-2.81)	2.02 (0.98-4.15)	-	-
	R-square	17%									
Quality of life	Univariate	0.90 **(0.83-0.98)**	0.94 (0.79-1.12)	1.02 (0.86-1.21)	1.18 (0.99-1.41)	1.32 **(1.12-1.58)**	0.96 (0.81-1.14)	0.96 (0.79-1.18)	1.08 (0.85-1.38)	-	-
	Multivariate	0.84 **(0.77-0.92)**	0.99 (0.86-1.16)	0.97 (0.82-1.14)	1.48 **(1.25-1.78)**	1.24 (0.96-1.60)	0.99 (0.80-1.22)	0.92 (0.23-1.17)	1.10 (0.83-1.46)	-	1.26 **(1.08-1.49)**
	R-square	31%									
Health distress	Univariate	1.15 **(1.06-1.26)**	1.09 (0.91-1.31)	1.02 (0.85-1.22)	1.04 (0.86-1.26)	1.16 (0.96-1.41)	1.11 (0.91-1.34)	0.93 (0.76-1.15)	0.99 (0.78-1.27)	-	-
	Multivariate	1.16 **(1.06-1.26)**	1.10 (0.92-1.32)	1.00 (0.82-1.21)	1.21 (0.97-1.49)	1.29 **(1.04-1.59)**	1.14 (0.89-1.45)	1.00 (0.76-1.32)	1.10 (0.80-1.50)	-	-
	R-square	13%									
Physical health summary score	Univariate	1.02 (0.94-1.09)	1.05 (0.90-1.22)	0.99 (0.85-1.15)	1.07 (0.97-1.25)	1.14 (0.97-1.33)	1.04 (0.90-1.22)	0.99 (0.84-1.17)	0.90 (90-1.09)	-	-
	Multivariate	0.95 (0.87-1.03)	1.22 (**1.03-1.45**)	0.98 (0.83-1.15)	1.18 (0.99-1.41)	1.03 (0.81-1.30)	1.00 (0.81-1.23)	1.15 (0.87-1.52)	0.89 (0.68-1.16)	0.68 **(0.49-0.95)**	1.22 **(1.04-1.43)**
	R-square	15%									
Mental health summary score	Univariate	1.04 (0.97-1.11)	1.03 (0.90-1.16)	1.01 (0.90-1.15)	1.04 (0.91-1.19)	1.16 **(1.02-1.33)**	1.05 (0.92-1.20)	0.96 (0.84-1.12)	1.01 (0.85-1.20)	-	-
	Multivariate	1.04 (0.98-1.11)	1.04 (0.91-1.18)	1.00 (0.87-1.15)	1.18 **(1.01-1.32)**	1.27 **(1.09-1.48)**	1.08 (0.90-1.29)	0.99 (0.81-1.21)	1.05 (0.84-1.32)	-	-
	R-square	9%									
Visual analogue scale	Univariate	0.95 **(0.91-0.98)**	1.01 (0.93-1.08)	0.97 (0.90-1.04)	0.92 (0.90-1.05)	1.24 **(1.16-1.32)**	1.03 (0.96-1.12)	0.93 (0.86-1.01)	1.00 (0.90-1.11)	-	-
	Multivariate	0.92 **(0.89-0.96)**	1.08 **(1.01-1.16)**	0.99 (0.92-1.05)	1.11 **(1.03-1.19)**	1.22 **(1.10-1.34)**	1.00 (0.92-1.09)	0.98 (0.87-1.09)	0.94 (0.84-1.05)	0.87 **(0.75-0.99)**	1.08 **(1.01-1.15)**
	R-square	41%									

The following were reference groups: private hospital, women, HIV negative, starting treatment patient category, and 18-24 years age group. MOS = Medical Outcomes Survey, HRQoL = Health-related quality of life

In multivariable analysis after adjusting for sex, HIV status, age, and patient category, patients who were enrolled from the public hospital had significantly lower HRQoL scores for perceived health (0.78 (95% confidence interval (CI); 0.64-0.95)), quality of life (0.84 (95% CI; 0.77-0.92)), and VAS subscales (0.92 (95% CI; 0.92-0.96)) relative to patients from the private hospital (Table 4). However, patients from the public hospital had significantly higher HRQoL scores (1.15 (95% CI; 1.06-1.26)) for health distress relative to patients from the private hospital. Patients who completed an 8 months course of TB therapy had significantly higher HRQoL scores for perceived health subscale (1.93 (95% CI; 1.19-3.13)), health distress subscale (1.29 (95% CI; 1.04-1.59)) and mental health summary scores (1.27 (95% CI; 1.09-1.48)) relative to patients that were starting therapy.

Further in multivariable analysis (Table 4), patients who completed 8 months of TB therapy among patients who were recruited from the public hospital had a significant increase in HRQoL scores for QoL subscale (1.26 (95% CI; 1.08-1.49)), physical health summary score (1.22 (95% CI; 1.04-1.43)), and visual analogue scale (1.08 (95% CI; 1.01-1.15)) relative to patients who were recruited from the private hospital and had completed 8 months of TB therapy. Men of 35 to 44 years in age were associated with significantly lower HRQoL scores for physical health summary score (0.68 (95% CI; 0.49-0.95)) and visual analogue scale (0.87 (95% CI; 0.75-0.99)) relative to women of the same age group. For the QoL scores for HIV positive relative to HIV negative patients were not significantly different for all the MOS subscales and the VAS scale (Tables 3 and 4).

## Discussion

We performed the feasibility and reliability of the MOS to measure HRQoL among HIV positive and HIV negative patients with pulmonary TB receiving care at public and private hospitals in urban, Uganda. The key finding in this study of 133 patients with pulmonary TB is that the MOS in measuring HRQoL performed well on the psychometric indicators and this instrument appears to be an effective tool for evaluating HRQoL among TB patients. The scale demonstrated acceptable internal consistency among TB patients with different stages of treatment. Evaluation of constructs revealed remarkable differences in the functional status and well-being among TB patients at different stages of treatment, hospital settings, and gender. However, no differences were seen by HIV status.

Findings in this study suggest that TB patients have poor HRQoL at the time of diagnosis and this impression appear to be marked among patients attending public compared to private health institutions particularly for perceived health, bodily pain, role function, and health distress. However, patients receiving care from public health institution had substantial relative increase in HRQoL scores for QoL subscale, physical health summary score, and visual analogue scale following TB therapy compared to patients who received care from private institution. This suggest that patients receiving care in public health institutions appear to catch-up in HRQoL to those receiving care in private institutions and that TB therapy improves HRQoL regardless of treatment setting.

In this study, men of older age had poor HRQoL score relative to women of similar age group suggesting that men were sicker compared to women. This probably reflects on the health seeking habits for men which could be that by the time men present, their disease is advanced. We found no differences in HRQoL scores by HIV status probably because HIV impacts minimally on HRQoL among patients with TB; however, this requires further evaluation in prospective studies and after teasing the effect of HIV disease severity.

Results of the psychometric testing of the MOS in a population of patients with pulmonary TB in the present study were similar to prior studies that culturally adapted it into the local language- Luganda in Uganda among HIV-infected women [7]. The reliability coefficients for the MOS subscales in prior studies among HIV-infected women were above 0.70 except for role functioning at 0.43 and vitality at 0.64. Similarly in the present study, all MOS subscales had coefficients above 0.70 except role functioning that had 0.65. Further evidence for validity of our findings is shown by the high correlation of each dimension of the MOS with the dimension measured concurrently using the VAS.

There is paucity of research on HRQoL among TB patients in the African population, and particularly few have evaluated HRQoL using generic standardized or disease-specific quality of life instruments. The score profiles in our study population were quite similar to those reported in a cross-sectional study from South Africa [17]. For example, the mental health score was 55.6 ± 12.8 for the South African study compared to 61.6 ± 25.5 for the present study. This suggests that HRQoL may be affected similarly by TB disease across cultural contexts. Compared to the South African study, the major strengths with our study is the heterogeneity of the study population that included patients from different hospital settings, patients at different stages of TB therapy, HIV positive, and HIV negative. Thus our study findings are generalizable to a wide-range of patients particularly countries in sub-Saharan Africa with a high burden of TB and HIV.

Our study was not without limitations including lack of test re-test reliability for us to comment on the stability of the MOS HRQoL scores across time. In addition, the study design was cross-sectional in nature and thus the associations may not be causal. Further, our findings were limited by lack of data on severity of HIV disease which might affect HRQoL scores [7]. Nevertheless, our findings may provide insight to the future predictive validity of the study instrument among TB patients because participants were enrolled at different stages of treatment.

In conclusion, this study provides evidence that the MOS instrument is a valid and reliable measure of HRQoL among TB patients and can be used in a wide variety of study populations and settings. Further, findings revealed that treatment improved HRQoL among TB patients. However, there were differences in HRQoL among TB patients by hospital settings, and by gender among older patients.

## Competing interests

The authors: HMB, DN, AK, and EM declare that they have no competing interests.

## Authors' contributions

HMB conceived the study; participated in its design, coordination, statistical analysis, and drafted the manuscript. DN participated in the design of the study, critical review of the manuscript, and final approval of the version to be published. AK participated in the design of the study and critical review of the manuscript. EM participated in the design of the study, statistical analysis, and critical of the manuscript. All authors have read and approved the final manuscript.

## Authors' information

HMB, MBChB, MPH currently a PhD student at Case Western Reserve University

DN, PhD, Professor at Case Western Reserve University, Department of Epidemiology and Biostatistics

AK, MBChB, DCH, M.S., PhD Lecturer Clinical Epidemiology Unit College of Health Sciences, Makerere University

EM, MBChB, M.MED, M.S. Lecturer Department of Paediatrics & Child Health, College of Health Sciences, Makerere University
